# Stability and Assembly Mechanisms of Butterfly Communities across Environmental Gradients of a Subtropical Mountain

**DOI:** 10.3390/insects15040230

**Published:** 2024-03-27

**Authors:** Fanyu Wei, Tingting Xie, Chengyong Su, Bo He, Zufei Shu, Yingming Zhang, Zhishu Xiao, Jiasheng Hao

**Affiliations:** 1 College of Life Sciences, Anhui Normal University, Wuhu 241000, China; weifanyu1996@163.com (F.W.); tingtingxie2021@ioz.ac.cn (T.X.); such@ahnu.edu.cn (C.S.); hebo90@ahnu.edu.cn (B.H.); 2 Key Laboratory of Zoological and Systematics and Evolution, Institute of Zoology, Chinese Academy of Sciences, Beijing 100045, China; 3 Guangdong Chebaling National Nature Reserve Administration Bureau, Shaoguan 512500, China; szfcbl@163.com (Z.S.); cblbhq@163.com (Y.Z.); 4 State Key Laboratory of Integrated Management of Pest Insects and Rodents in Agriculture, Institute of Zoology, Chinese Academy of Sciences, Beijing 100045, China

**Keywords:** butterfly, mountain ecosystem, biodiversity, community stability, community assembly mechanisms, phylogenetic placement

## Abstract

**Simple Summary:**

Biodiversity research on mountain ecosystems is mainly focused on vertebrates and vascular plants; therefore, we are still far from understanding the general diversity patterns of mountain insects. In this study, we surveyed and recorded butterflies in a typical subtropical mountain in the Chebaling National Nature Reserve, Guangdong Province, China, through grid-based monitoring across the entire region for two years, and then investigated their taxonomic and phylogenetic diversities. The results show that taxonomic diversity played a considerable role in mediating the effects of environmental changes on stability; stochastic processes are dominant in governing the composition of butterfly assemblages; increasing selective pressure mainly caused by abiotic factors enhanced the heterogeneous selection processes at higher elevations; placement trees could improve the accuracy compared to barcode trees in community phylogenetic analyses.

**Abstract:**

Mountain ecosystems harbor evolutionarily unique and exceptionally rich biodiversity, particularly in insects. In this study, we characterized the diversity, community stability, and assembly mechanisms of butterflies on a subtropical mountain in the Chebaling National Nature Reserve, Guangdong Province, China, using grid-based monitoring across the entire region for two years. The results showed that species richness, abundance, and Faith’s phylogenetic diversity decreased with increasing elevation; taxonomic diversity played a considerable role in mediating the effects of environmental changes on stability. Moreover, our results showed that stochastic processes are dominant in governing the assembly of butterfly communities across all elevational gradients, with habitats at an elevation of 416–580 m subjected to the strongest stochastic processes, whereas heterogeneous selection processes displayed stronger effects on the assembly of butterfly communities at 744–908 m, 580–744 m, and 908–1072 m, with abiotic factors inferred as the main driving forces. In addition, significant differences were detected between the barcode tree and the placement tree for the calculated β-NTI values at 416–580 m. Overall, this study provides new insights into the effects of environmental change on the stability and assembly of butterflies in Chebaling, which will be beneficial for biodiversity conservation and policy development.

## 1. Introduction

Terrestrial biogeographic regions occupy little of the Earth’s land surface [1,2,3], and mountainous ecosystems harbor the richest and most diverse terrestrial biodiversity [4]. To date, biodiversity research on mountain ecosystems has mainly focused on vertebrates and vascular plants. We are still far from understanding the general diversity patterns of mountain insects, which are the most species-rich group in the animal kingdom and play an important role in ecosystems and human life [5,6,7].

Individual populations of species do not exist alone, but instead interact to form complex communities with a higher or lower level of stability [8]; thus, they could remain unchanged in response to projected changes in the environment. There is growing interest in predicting the relationships between the stability and complexity of communities, and a previous study has suggested that they are strongly correlated [9]; however, little is known about how complexity affects stability under environmental change scenarios [10,11]. Along with complexity, stability is also potentially mediated by diversity, such as taxonomic and phylogenetic diversity, and a previous study suggested that a higher diversity increases stability by increasing the number of species that respond differently to environmental changes [12]; however, a recent study has indicated that communities with more diversity are not always more stable against environmental changes [13], while another study showed that a higher diversity may also imply a higher number of species stressed or damaged by environmental changes [14].

The fundamental theories of species coexistence, namely, the niche-based theory and neutral theory, could provide a foundation for our understanding of the assembly mechanisms of community composition, as well as the responses of communities to environmental changes [8,15,16]. However, the relative contributions of different processes, whether deterministic (such as homogeneous and heterogeneous selection), or stochastic (such as dispersal limitation, homogenizing dispersal, and undominated), may vary across different organisms, environmental conditions, and ecosystems. For example, in temperate steppes, deterministic processes dominate plant community assembly, whereas stochastic processes strongly shape soil fungal community assembly [17,18]; in Eurasian steppes, stochastic processes played an important role in the assembly of soil microbial communities [19].

Numerous phylogeny-based ecological studies have been published in the recent decades since Cam Webb’s work on community phylogenetics [20], in which the degree of relatedness among species in ecological communities was calculated using branch lengths in a phylogenetic tree. Historically, these studies have focused on DNA barcode trees, which are constructed using either single- or multiple-gene sequence data, and several studies have indicated that barcode trees may have promising performance for inferring community phylogenetic structures [21,22,23]. Nonetheless, there is a growing awareness that barcode trees are still problematic, compared with more robust phylogeny trees (e.g., placement trees, which place the barcodes on published phylogenies), whether they are robust or not, when considering the assembly mechanisms of communities [24], especially in species-rich geographical regions [25].

Global environmental changes have become increasingly apparent, accompanied by sharp increases in human activity since the mid-20th century [26], leading to an intense decline in insect populations worldwide [27]. Therefore, it is important to investigate diversity patterns and clarify the mechanisms underlying the stability and assembly of insect communities, as well as their responses to environmental changes. The requisite long-term monitoring data of community composition required for such assessment are especially lacking in biodiversity hotspots [28], which are recognized for their potential to provide basic, but vital, data for the aforementioned assessment, and ultimately promote their conservation and management. As a representative group of insects, butterflies are considered one of the most effective bioindicators for ecological monitoring because of their high species richness and well-characterized taxonomy [29]. Moreover, butterflies are remarkably sensitive to climatic and environmental changes because they depend exclusively on host plants and environmental niches [30].

Here, we surveyed and recorded butterflies in a typical subtropical mountain in the Chebaling National Nature Reserve, Guangdong Province, China, through grid-based monitoring across the entire region for two years, and then investigated their taxonomic and phylogenetic diversities. Based on these data, this study aimed primarily to address the following questions: (1) what are the patterns of butterfly diversity in Chebaling and what are their best environmental predictors; (2) how do complexity and diversity affect stability under environmental change scenarios; (3) the relative importance of deterministic and stochastic processes in shaping the composition of butterfly communities; and (4) whether we can achieve the same results in phylogenetic analyses using barcode and placement trees.

## 2. Materials and Methods

### 2.1. Study Area, Butterfly Survey and Environmental Factors

**Figure 1:**
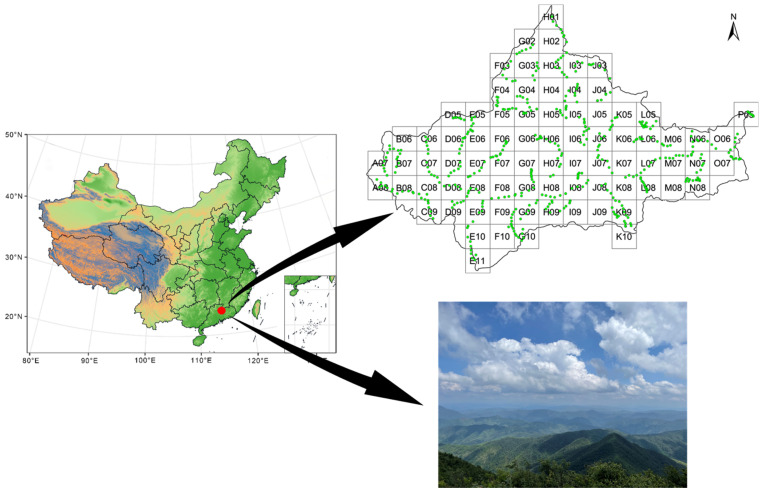
The location of sampling sites in the Chebaling National Nature Reserve, Guangdong Province, China. Green dots are the monitoring routes.

The Chebaling National Nature Reserve (Figure 1) is located southeast of Shaoguan City, Guangdong Province, China (114°09′04″–114°16′46″ E, 24°40′29″–24°46′21″ N), representing a typical transitional zone from a southern subtropical to central subtropical area and covering a total area of 75.45 km^2^ with elevations ranging from 330 to 1256 m above sea level. This reserve is covered by subtropical evergreen broadleaf forests with its climate regime having a subtropical monsoon climate. The annual average temperature and precipitation are approximately 19.6 °C and 2126 mm, respectively. Additionally, the landform of this area is characterized by mountains that are typical of the South China fold system and exhibit a complex topography.

In 2021 and 2022, we investigated the diversity and distribution of butterflies in the entire Chebaling reserve eight times (April, May, June, and September in each year). Considering the distributional narrowness of some rare species, and to ensure adequacy and representativeness of monitoring, we first established a grid-based butterfly diversity monitoring network by dividing the entire area into 80 grids with a size of 1 × 1 km. Within each grid, a 1-km transect was established, which was surveyed eight times over two years. During surveys, one observer and one recorder walked at a constant pace of 1–1.5 km/h along the transect and recorded and identified all butterflies within 5 m of the transect line. When identification was not possible in the field, photographs were taken, and identifications could be made later with the aid of guide [31]. Partial butterfly samples of 237 species were collected and preserved in 99.5% ethanol for fixation and stored at −20 °C for genomic DNA study in the laboratory of Insect Diversity of Evolution, at the College of Life Sciences, Anhui Normal University.

Five environmental factors, elevation, slope, canopy density, enhanced vegetation index (EVI), and distance to the nearest settlement (NEAR_DIST, anthropogenic perturbations), were used in this study and were determined by Xiao [32]. The elevation and slope were extracted directly from ASTER GDEM (https://lpdaac.usgs.gov, accessed on 23 March 2024), with their mean values being obtained in each sampling grid; the canopy density was obtained by calculating the annual forest inventory data of the reserve area; the mean value of EVI was extracted directly from Landsat-8 based on cloud-free data for 2017; the NEAR_DIST was obtained from the ‘Gaofen-1’ satellite and then analyzed using ArcGIS (version 10.4).

### 2.2. Extraction and Sequencing of COI DNA

Individual legs of all 237 species stored in absolute ethanol at −20 °C were used for the molecular study; total genomic DNA was extracted with the QIAGEN DNeasy Blood and Tissue Kit (Hilden, Germany) following the manufacturer’s instructions. The *COI* DNA barcode region (~650 bp) was amplified using the universal primer pairs, LCO1490 and HCO2198 [33]. When *COI* sequences were not successfully generated, one of the alternative primer pairs, LepF1 and LepR1 [34], were used to amplify the target gene. Polymerase chain reactions (PCR) were performed in 30 μL solutions, following the protocol of Wang et al. [35]. PCR products were sequenced at Shanghai Sangon Biotechnology, Co., Ltd. (Shanghai, China), and the quality was checked using SeqMan [36] and MEGA (version 7.0) [37].

### 2.3. Phylogenetic Placement Tree Reconstruction

To improve the phylogenetic topology of *COI*-only matrices with limited information content, a phylogeny was constructed for the DNA barcodes, integrating information from the backbone tree, according to the constraint method described by Chesters [38]. In the procedure, for setting the backbone topology, we selected the butterfly phylogeny publicly available from insectphylo.org (https://insectphylo.org/lepidoptera/; accessed on 29 July 2023) [39], followed by an additional set of soft constraints for the *COI*s corresponding to any monophyletic taxa observed in the source trees. The inference was conducted with three partitions corresponding to each codon position and binary partitions according to the taxon constraints inferred from the analysis of backbone tree. Finally, a phylogenetic placement tree was constructed using RAxML (version 8.2.10) (command line option−g) [40].

### 2.4. Diversity Patterns

Prior to analyses, data from the eight survey times were pooled at the grid level. The taxonomic and phylogenetic diversity patterns of butterflies were first analyzed using species richness (SR) and abundance as measures of taxonomic alpha diversity [41], and then Faith’s phylogenetic diversity (PD), which sums all branch lengths in a phylogeny that connects species in a community [42], as well as the mean nearest taxon distance (MNTD) which is the average shortest phylogenetic distance for each species to its closest relative in a community [43], to evaluate phylogenetic alpha diversity. Variations in the taxonomic and phylogenetic diversity of butterflies in relation to elevation were assessed using Pearson’s correlation coefficient, and the most important drivers of SR, abundance, PD, and MNTD correlated with the five environmental factors were identified using a random forest model [44]. Additionally, the value of species richness is often underestimated when based on observed species richness, which may misinform our understanding of diversity patterns. To account for this, we used the first three Hill numbers to estimate species richness (*q* = 0), the exponential of Shannon’s entropy (*q* = 1; referring to Shannon diversity), and the inverse of Simpson’s concentration (*q* = 2; referring to Simpson diversity) of the butterfly communities [45].

To assess dynamics in the composition of the butterfly communities, the total taxonomic and phylogenetic beta diversities were calculated using the pair-wise Bray–Curtis and PhyloSor dissimilarity indices, respectively, and then the calculated diversity values were partitioned into nestedness and turnover components following two approaches described by Baselga et al. (2010) and Cardoso et al. (2014) (i.e., BAS and POD methods) [46,47,48]. All statistical analyses were performed using R (version 4.2.2) [49].

### 2.5. The Effect of the Complexity and Diversity on Stability under Environmental Changes

The degree of complexity of butterfly communities can be quantified using cohesion analysis by calculating the total abundance-weighted pairwise correlations of every taxon in a given community, with cohesion values illustrating positive or negative species interactions and reflecting the degree of cooperative behavior and competitive interactions [50]. In addition, the stability of butterfly communities was evaluated by the average variation degree (AVD), which was calculated using the degree of deviation from the mean value of the relative abundance of normally distributed species at different elevation levels [51].

The direct, indirect, or both effects of environmental changes on community stability were quantified using piecewise structural equation modeling (piecewiseSEM) [52]. Prior to SEM analyses, the ‘hier.part’ package in R [53] was used to identify the most important predictors in influencing community stability among the five environmental factors. The three most important factors were then converted into a composite variable to simplify the initial model. Three independent SEM models were used to evaluate the influence of complexity and diversity as possible mediators of stability: (1) complexity and diversity were both included in the full model; (2) only diversity was added to a separate model; (3) only complexity was added to a separate model. Furthermore, linear mixed-effects models were used to provide the ‘marginal’ and ‘conditional’ contributions of predictors in driving community stability, avoiding the potential influences of different sampling sites. Fisher’s C-test was used to assess the goodness of all modeling results. We then modified our models according to the significance (*p* < 0.05) and the goodness of the model (0 ≤ Fisher’s C/df ≤ 2 and 0.05 < *p* < 1.00).

### 2.6. Assembly Mechanisms of Butterfly Communities

To evaluate the potential effects of neutral processes on the assembly of butterfly communities in the nature reserve, we used the Sloan neutral community model (NCM), which predicts that the taxa abundant in the metacommunity will be widespread because of their stronger dispersal ability among different sampling sites, whereas rare taxa are more likely to be lost at different sites because of ecological drift [54,55]. The estimated ‘*m*’ represents the migration rate of the individual taxon, with its higher value indicating a lower probability for the dispersal limited of communities. The parameter R^2^ indicates the goodness of fit to the neutral model.

The relative importance of niche-based and neutral processes in shaping the composition of butterfly communities, was determined using the null model approach [56], which is based on the assumption that closely related taxa are ecologically similar, and thus, the Mantel correlogram should be used to test the phylogenetic signal before null model analysis. According to the null model approach, the niche-based and neutral processes can be distinguished by calculation of standardized phylogenetic turnover between communities, quantified by the beta nearest taxon index (β-NTI). Subsequently, the combination of β-NTI values and the Bray–Curtis-based Raup–Crick index (RCbray) were used to further estimate the relative contributions of assembly processes (homogeneous selection, heterogeneous selection, dispersal limitation, homogenizing dispersal, and undominated processes). In addition, the Kruskal–Wallis test was used to evaluate whether there are significant differences in the β-NTI values across different elevational gradients (416–580 m, 580–744 m, 744–908 m, and 908–1072 m).

### 2.7. Phylogenetic Barcode Tree Reconstruction

To investigate whether the results of the phylogenetic analyses, including diversity assessment and community assembly mechanisms, are dependent on the availability of a robust reference phylogeny, a maximum-likelihood barcode tree was constructed based on mitochondrial *COI* sequences only. The *COI* alignment was performed using MAFFT software (version 7.490) [57] with the L-INS-i parameter, and aligned sequences were trimmed to remove discrepant sites using Gblocks software (version 0.91b) [58] and manually proofread using BioEdit software (version 7.0.9.0) [59]. The best-fit partitioning schemes and substitution models used herein were recommended by ModelFinder and implemented in IQ-TREE [60]. We then searched for the most likely tree in IQ-TREE (version 2.1.3) [61], with 1000 iterations for ultrafast bootstrap approximation [62]. Pearson’s correlation coefficient was used to examine the correlation of phylogenetic diversity between the barcode and placement trees, and the Wilcoxon test was used to test whether there were significant differences in the β-NTI values obtained from the two phylogenetic tree constructions.

## 3. Results

### 3.1. Diversity Patterns

**Figure 2:**
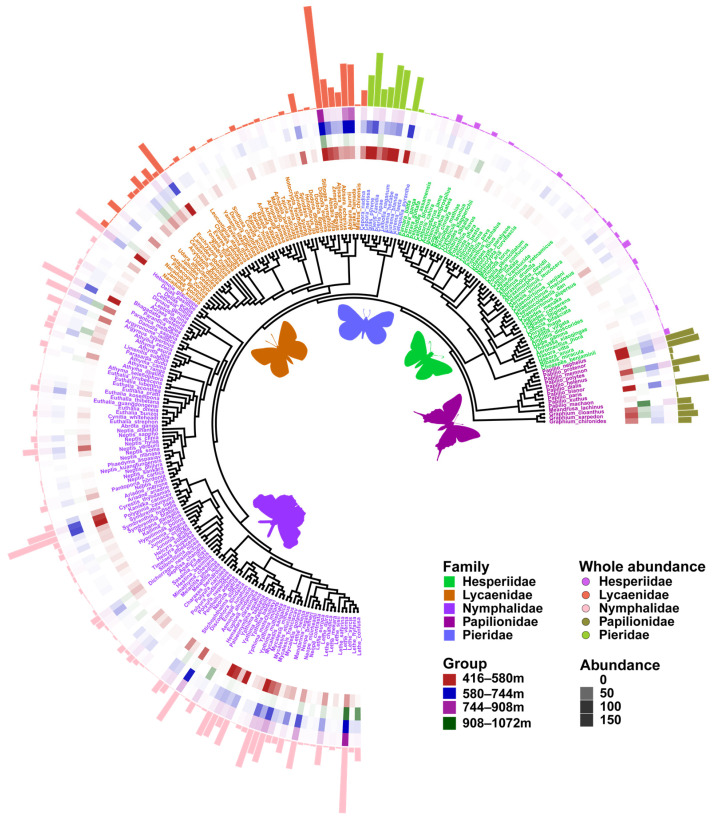
Illustration of mapping and visualizing associated data on phylogeny, species abundance distributions were aligned to the tree. The placement tree from maximum likelihood (ML) analysis was constructed according to the constraint method and visualized using ggtree and ggtreeExtra. Different families are labeled with different colors. The heatmap (inner rings) indicates the relative abundance of each species in different elevational gradients; the histogram (outer ring) in the outer layer depicts the relative abundance of each species.

In total, 6905 butterflies of 237 species (Figure 2) from five families and 129 genera were observed in the Chebaling nature reserve. The butterfly species list is presented in Table S1. Both species accumulation curves [63] and sampling coverage assessment [64] indicated that almost all known butterfly species known to be in the area were detected in Chebaling (Figure S1; Table S2).

**Figure 3:**
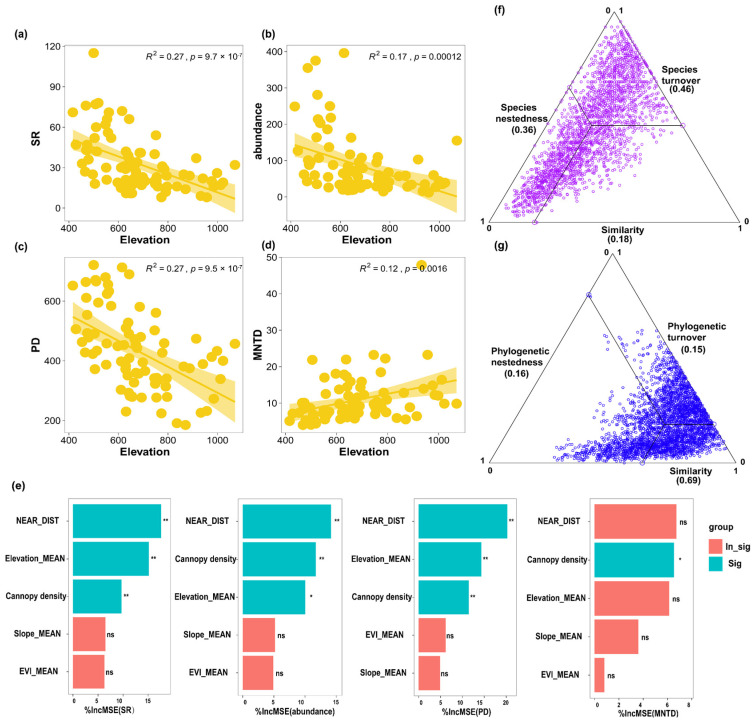
Variations in (**a**) species richness (SR), (**b**) abundance, (**c**) Faith’s phylogenetic diversity (PD), and (**d**) mean nearest taxon distance (MNTD) of butterflies along elevation in Chebaling; regression lines refer to the significant relationship between the two variables detected through linear models and shading areas associated with the lines represent the 95% confidence interval; (**e**) the importance of the five environmental factors (analyzed using a random forest model) in driving SR, abundance, PD, and MNTD, significance levels: *: *p* < 0.05, **: *p* < 0.01, and ns: *p* > 0.05; decomposition analysis of (**f**) taxonomic beta diversity, and (**g**) phylogenetic beta diversity.

For alpha diversity, SR, abundance, and PD were significantly negatively related to elevation (Figure 3a–c), with NEAR_DIST (*p* < 0.01) being the most important explanatory variable (Figure 3e). Moreover, the first three Hill numbers (*q* = 0, *q* = 1, and *q* = 2) also declined with increasing elevation (Figure S2a,c), regardless of the focus on rare or common species. MNTD was significantly positively related to elevation (Figure 3d). Regarding taxonomic beta diversity, the BAS analysis showed that the turnover component was the main contributing factor to the difference in the composition of the butterfly community (46%), followed by the nestedness component (36%) (Figure 3f); the POD analysis showed that the nestedness component was the main contributing factor to the difference in the composition of the butterfly community (44%), followed by the turnover component (43%). When evolutionary history, namely phylogenetic beta diversity, was considered, the BAS analysis showed that the nestedness component contributed slightly more (16%) than the turnover component (15%) (Figure 3g); the POD analysis showed that the nestedness component contributed more (48%) than the turnover component (17%).

### 3.2. The Effect of Complexity and Diversity on Stability under Environmental Changes

**Figure 4:**
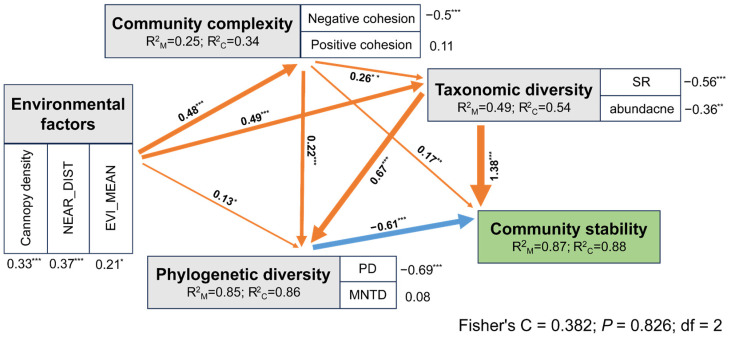
The direct and indirect effects of environmental factors, community complexity, taxonomic diversity, and phylogenetic diversity on the responses of community stability using piecewise SEM. The environmental factors, community complexity, taxonomic diversity, and phylogenetic diversity were changed to composite variables. Numbers adjacent to measured variables are their coefficients with composite variables. Numbers adjacent to arrows are path coefficients are the directly standardized effect size of the relationship. The thickness of the arrow represents the strength of the relationship. The conditional (C) and marginal (M) R^2^ represent the proportion of variance explained by all predictors without and with accounting for random effects of ‘sampling site’. Significance levels of each predictor are * *p* < 0.05, ** *p* < 0.01, *** *p* < 0.001.

In the SEM analyses, the effects of environmental factors on community stability were indirect through the alteration of community complexity and diversity (taxonomic and phylogenetic diversity), and the models were not suitable (*p* < 0.05) when the direct effects were added. Complexity and taxonomic diversity have direct positive effects on stability, whereas phylogenetic diversity has direct negative effects. In addition to the direct effect, complexity indirectly affected the stability by directly affecting taxonomic or phylogenetic diversity (Figure 4). When the direct and indirect effects of individual factors were considered together, three of the four factors retained in the model positively affected stability (Figure S4b). Moreover, when combining complexity and diversity (taxonomic and phylogenetic diversity), the model explained a large proportion of community stability (82.6%) (Figure 4), and when considering only diversity (taxonomic and phylogenetic diversity), the model explained 56.8% of the variation in stability (Figure S4c); when only considering complexity, the model fit was unacceptable (*p* < 0.05) (Figure S4d).

### 3.3. Assembly Mechanisms of Butterfly Communities

**Figure 5:**
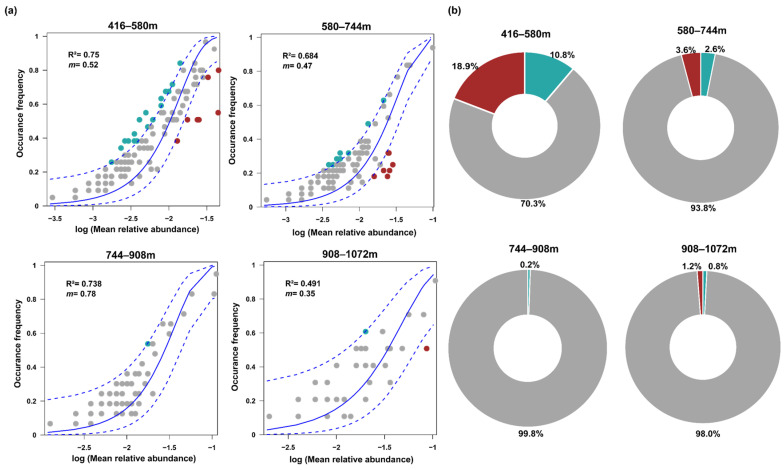
(**a**) The effects of random dispersal and ecological drift on the assembly of butterfly communities at different elevations through neutral model analysis. R^2^ indicates the goodness of fit to the neutral model. *m* indicates the estimated migration rate. The solid blue lines indicate the best fit to the neutral model and dashed blue lines represent 95% confidence intervals around the model prediction. (**b**) The relative abundance of the over-represented (light blue), neutrally distributed (grey) and under-represented species (red) in the butterfly communities.

Overall, our NCM analyses showed that the frequency of butterfly occurrence at different elevational gradients had a moderate fit to the neutral model, except for the elevational gradient of 908–1072 m, in the order of 416–580 m (R^2^ = 0.75) > 744–908 m (R^2^ = 0.738) > 580–744 m (R^2^ = 0.684) > 908–1072 m (R^2^ = 0.491). As for the estimated migration rate ‘*m*’, 744–908 m had the highest migration rate (*m* = 0.78), whereas 908–1072 m had the lowest (*m* = 0.35) (Figure 5a). Furthermore, the ratios of relative abundances of neutrally distributed butterflies were 70.3, 93.8, 99.8, and 98% at 416–580 m, 580–744 m, 744–908 m, and 908–1072 m, respectively (Figure 5b).

**Figure 6:**
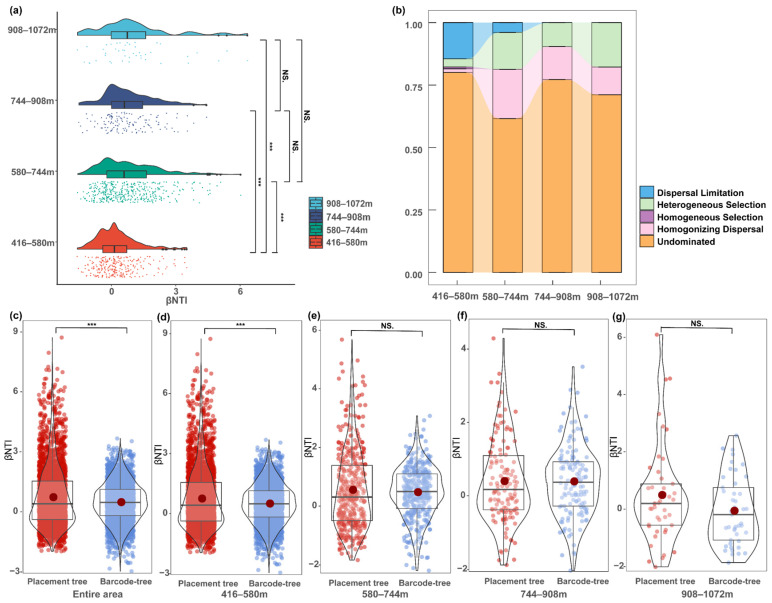
(**a**) The significance of the difference in the values of beta nearest taxon index (βNTI) across different elevational gradients (416–580 m, 580–744 m, 744–908 m, and 908–1072 m) tested by Kruskal–Wallis; (**b**) the relative contribution of each ecological process to butterfly community assembly; the significance of the difference between the values of β-NTI obtained from placement tree and barcode tree tested by Wilcox in the (**c**) entire area, at (**d**) 416–580 m, (**e**) 580–744 m, (**f**) 744–908 m, and (**g**) 908–1072 m communities; significance levels: ***: *p* < 0.001 and NS.: *p* > 0.05.

Testing for phylogenetic signals using Mantel correlograms revealed significant positive correlations between differences in environmental factors and phylogenetic distances (*p* < 0.05) across relatively short phylogenetic distances (Figure S5). Thus, the underlying ecological processes could be inferred from the analyses of phylogenetic turnover, and the relative importance of different ecological processes was assessed using null model analysis. First, the results showed that highly significant differences (*p* < 0.01) were detected for the β-NTI values for communities at 416–580 m, 580–744 m, 744–908 m, and 908–1072 m) (Figure 6a). Second, at 416–580 m, the stochastic process of undominated (80.07%) was responsible primarily for the assembly of the butterfly communities, followed by dispersal limitation (14.49%), while at 580–744 m, the stochastic process of undominated (61.58%) was responsible primarily for the assembly of butterfly communities, followed by homogenizing dispersal (19.7%). At 744–908 m, the stochastic process of undominated (77.21%) was responsible primarily for the assembly of the butterfly communities, followed by homogenizing dispersal (13.24%); at 908–1072 m, the stochastic process of undominated (71.11%) was responsible primarily for the assembly of the butterfly communities, followed by heterogeneous selection (17.78%). These results indicate that stochastic processes play a dominant role in the assembly of butterfly communities across all elevational gradients. Third, the heterogeneous selection processes exerted greater effects on the assembly of butterfly communities at relatively higher elevations (580–744 m, 744–908 m, and 908–1072 m) than at lower elevations (416–580 m), and abiotic factors (the five environmental factors mentioned) (*r* = 0.22, *p* < 0.01) rather than biotic factors (negative cohesion and positive cohesion) (*r* = 0.15, *p* < 0.01) enhanced the heterogeneous selection processes at higher elevations.

### 3.4. Phylogenetic Analyses Using Barcode and Placement Trees

Our results showed that both the PD estimated using the barcode tree and that of the placement tree (Figure 2) were both significantly correlated with taxonomic diversity (Figure S6). The β-NTI values derived from the two phylogenetic trees showed highly significant differences (*p* < 0.01) in the entire community and those at 416–580 m (Figure 6c,d), whereas no significant differences were observed among the communities at 580–744 m, 744–908 m, and 908–1072 m (Figure 6e–g).

## 4. Discussion

### 4.1. Diversity Patterns

Our grid-based butterfly monitoring data highlighted the changes in butterfly diversity along elevational gradients, that is, the alpha diversity indices of SR, abundance, and PD all strongly decreased with increasing elevation in the Chebaling area, which has also been found in previous studies in other areas [65,66]. These decreased patterns along the elevational gradients found previously are not only true for the butterflies in this study, but also true for other insect taxa, such as the noctuid moths, green lacewings, and beetles [67,68,69]. NEAR_DIST was inferred to be the most important explanatory variable for the diversity patterns of the butterflies mentioned above, with decreased values fostering SR, abundance, and PD (Figure S3a,d,g). Our findings are consistent with those of some previous studies (e.g., Lyu et al., 2009) [70], although anthropogenic environmental degradation has recently become prevalent in mountain ecosystems [71]. Moreover, the declining temperature, decreasing resources, and habitat complexity are likely explanatory factors for the decreasing patterns of diversity in other studies [72,73,74].

Furthermore, similar to other studies that partitioned beta diversity into turnover and nestedness components [75,76], our BAS method showed that the turnover component was the main factor contributing to the differences in the composition of butterfly communities along the elevation gradient of the mountainous areas. This indicates that highland communities are not subsets of lowland communities, and that both environmental filters and dispersal limitations may have selected butterfly species along different elevational gradients [77]. However, our POD method showed that the nestedness component was the main factor contributing to the differences in the composition of butterfly communities, meanwhile, both BAS and POD methods indicated that the nestedness component was the main factor when evolutionary history was considered. This suggests that the BAS method can lead to an overestimation of the role of the turnover component [46], and phylogenetic beta diversity is not always as high as taxonomic beta diversity increases because of the similar evolutionary histories of different species [78].

### 4.2. The Effect of Complexity and Diversity on Stability under Environmental Changes

How environmental changes alter the relationships among community complexity, diversity, and community stability has gained attention in recent years. However, most related studies have focused on plant and microbiome communities [9,79,80], and studies on insect communities, especially butterflies remain limited.

Our results indicated that taxonomic diversity plays an important role in the stability of butterfly communities as a buffer against the impact of environmental changes, supporting the diversity–stability hypothesis of plant and microbial communities in previous studies [79,81,82,83]. In general, the most diverse communities are the most stable over time; that is, extreme environmental changes make the relative abundance of species highly variable over time, and the coexistence of a higher number of species that respond differently to environmental changes improves community stability by increasing the probability of survival under unfavorable environmental conditions [12]. In addition, communities with high phylogenetic diversity are expected to be more stable against environmental changes because of increased niche differentiation and complementary resources [84]. In general, phylogenetic diversity can be seen as representing the diversity of phylogenetically conserved functional traits, which may constitute a broader set of traits than is typically included in measures of functional diversity; however, there may be functionally important trait differences among species that are not explained in full by phylogenetic diversity [85]. We found that phylogenetic diversity had strong negative effects on stability, indicating that the butterfly communities in Chebaling are dominated by phylogenetically clustered functional groups [86]. Our result is generally in line with previous studies indicating that stability is often dependent on the functional traits of their dominant species [87,88].

A long-standing central ecological hypothesis suggests that complexity improves stability [10,89], and this has been increasingly reported in plant and microbial studies [9,90]. Our results show that complexity has a direct positive effect on stability. In addition to the direct effect, a high level of complexity could trigger high taxonomic diversity, and ultimately drive stability owing to cascading effects, namely the stability variations with environmental changes could result from complexity, and the relative abundance of different taxa significantly changed accordingly [90,91]. Moreover, we found that the piecewise SEM explained the highest variation in stability when complexity and diversity were combined. These results are consistent with those of recent studies that confirmed that the impact of environmental changes on stability was not exclusively mediated by diversity [92,93], but was also responsible for the alteration of stability by complexity.

### 4.3. Assembly Mechanisms of Butterfly Communities

Generally, deterministic and stochastic processes are recognized to simultaneously govern community assembly [16,94], and quantifying the relative contributions of deterministic and stochastic processes to community assembly is a central objective of ecological studies [94]. In this study, both the NCM and null model analyses suggested that stochastic processes primarily governed the assembly of butterfly communities across all elevational gradients, which is consistent with previous studies on archaeal communities in soil environments [95,96]. However, opposite patterns have also been observed in other studies (e.g., Zhang et al., 2018) [97]. Such discrepancies in community assembly are likely partially attributed to spatial-scale dependency, habitat diversity, and different populations [98,99,100]. Moreover, our results showed that the community at 416–580 m was subjected to the strongest stochastic processes, which might be due to anthropogenic perturbations and less environmental stress at low elevational gradients [101,102].

Empirical evidence suggests that the deterministic process includes both abiotic and biotic factors that influence the presence or absence and relative abundances of species [103], which may contribute to the community assembly process at higher elevations together [104]. Our findings showed that the deterministic process of heterogeneous selection displayed strong effects on the assembly of butterfly communities distributed at 580–744 m, 744–908 m, and 908–1072 m, in which environmental factors were more related to heterogeneous selection than biotic interactions. This finding provides strong evidence of the important role of environmental filtering at relatively high elevations [105]. However, these abiotic and biotic factors only partially explained the variations in the assembly of butterfly communities, which is consistent with numerous previous studies [16,106]. Such unexplained variations in community assembly can be attributed to unmeasured abiotic factors, biotic interactions, and stochastic processes [107,108]. Moreover, the ecological responses of different insects to environmental changes may vary widely due to differences in environmental plasticity. As habitat specialists, butterflies depend exclusively on host plants and environmental niches; thus, they lack environmental plasticity and are able to shift their community composition to cope with fluctuations in environmental conditions [109]. In this case, understanding the assembly mechanisms of butterfly communities across different elevational gradients is important for interpreting and predicting possible changes when they are exposed to several disturbances, and may help to better protect their biodiversity in the face of environmental changes.

### 4.4. Phylogenetic Analyses Using Barcode and Placement Trees

In recent decades, numerous studies have used phylogenetic analyses to investigate phylogenetic diversity and community assembly mechanisms. However, phylogenetic analyses can be affected by methodological, evolutionary, and environmental stresses [110], and in most cases, this scenario is intensified in species-rich regions. Our analyses showed that the type of phylogenetic reconstruction method used and environmental changes likely influenced the phylogenetic analyses of butterfly communities.

First, our results showed that the two PDs estimated using the barcode and placement trees were both correlated with taxonomic diversity. Thus, the use of a barcode tree may be sufficient when researchers focus only on the metric values of community phylogenetic diversity; for example, diversity patterns and the identification of areas with high PD values have been proposed for prioritized conservation [23,24,110,111,112].

Second, significant differences in β-NTI values of the entire community between barcode and placement trees were detected in this study, indicating that the use of only a barcode tree may be invalid in species-rich regions. Barcode trees, constructed using DNA barcodes, are constrained by a limited number of DNA barcodes, which may lack sufficient genetic variation to fully resolve phylogenetic relationships [113,114]. However, DNA barcodes have different rates of evolution owing to different ecological and evolutionary processes operating at different evolutionary time scales. For example, conserved DNA barcodes might provide important insights into the processes acting on long evolutionary time scales, whereas rapidly evolving barcodes might signal more recent speciation events [115]. Thus, it is usually difficult to determine the relative contribution of ecological processes to community assembly, and in the future, the more precise interpretations based on more robust phylogenetic trees are required to understand the real community assembly mechanisms [116].

Third, we found that there are significant differences in β-NTI values in the community at 416–580 m between barcode and placement trees. However, there were no major differences in the communities at 580–744 m, 744–908 m, and 908–1072 m. These findings indicated the considerable role of the environmental stress with elevation in phylogenetic analyses [110].

## 5. Conclusions

Our study showed that SR, abundance, and PD of butterflies in the Chebaling area decreased as elevation increased, with NEAR-DIST being inferred as their major driver, highlighting that taxonomic diversity played a critical role in stability as a buffer against the impact of environmental changes, and the increasing selective pressure mainly caused by abiotic factors rather than biotic factors enhanced the heterogeneous selection processes at higher elevations. Furthermore, our results demonstrated that placement trees could improve the accuracy compared to barcode trees in community phylogenetic analyses. These results will contribute to a better understanding of the susceptibility of butterfly communities to environmental changes and provide useful scientific criteria for relevant conservation and management actions.

## Data Availability

The data that support the findings of this study are available in Supplementary Materials.

## Supplementary Materials

The following supporting information can be downloaded at https://www.mdpi.com/article/10.3390/insects15040230/s1. Table S1. Checklist of identified butterflies in the Chebaling study area. Table S2. The sampling coverage was estimated using the ‘iNEXT’ package in R across all grids. Figure S1. Species accumulation curves of the observed butterfly samples in Chebaling. Figure S2. Variations of (a) Hill numbers (*q* = 0), (b) Hill numbers (*q* = 1), and (c) Hill numbers (*q* = 2) of butterflies along elevation in Chebaling, regression lines refer to the significant relationship between the two variables detected through linear models and shading areas associated with the lines represent the 95% confidence interval. Figure S3. Correlation between diversity indices and environmental factors (the most important drivers analyzed by Random Forest model). Relationships between NEAR_DIST and (a) species richness (SR), (d) abundance, (g) Faith’s phylogenetic diversity (PD), and (j) mean nearest taxon distance (MNTD); relationships between Elevation_MEAN and (b) SR, (f) abundance, and (h) PD; relationships between Cannopy density and (c) SR, (e) abundance, and (i) PD. Regression lines refer to the significant relationship between the two variables detected through linear models and shading areas associated with the lines represent the 95% confidence interval. Figure S4. (a) Visualization of the independent contributions of the different environmental factors to the community stability (result of R-function ‘hier.part’); (b) the direct, indirect, and total standardized effects of composite variables on community stability; (c) the direct and indirect effects of environmental factors, taxonomic diversity, and phylogenetic diversity on the responses of community stability using piecewiseSEM; (d) the direct and indirect effects of environmental factors and community complexity on the responses of community stability using piecewiseSEM. The environmental factors, community complexity, taxonomic diversity, and phylogenetic diversity were altered to composite variables. Numbers adjacent to measured variables are their coefficients with composite variables. Numbers adjacent to arrows are path coefficients are the directly standardized effect size of the relationship. The thickness of the arrow represents the strength of the relationship. The conditional (C) and marginal (M) R^2^ represent the proportion of variance explained by all predictors without and with accounting for random effects of ‘sampling site’. Significance levels of each predictor are * *p* < 0.05, ** *p* < 0.01, *** *p* < 0.001. Figure S5. Phylogenetic Mantel correlogram evaluating phylogenetic signal in the butterfly communities observed in this study. The plot relates Pearson correlation coefficients to phylogenetic distances classes. Significant correlations (*p* < 0.05; green dots) indicate significant phylogenetic signal but only across relatively short phylogenetic distances. Figure S6. Correlation among species richness (SR), abundance, Faith’s phylogenetic diversity (PD), and mean nearest taxon distance (MNTD). PD_col and MNTD_col were calculated using the barcode tree; PD_place and MNTD_place were calculated using the placement tree. Significance signs for correlation coefficients are marked in the circles: * means *p* < 0.05, ** means *p* < 0.01, *** means *p* < 0.001.
