# Supplementary material for: Stability and Assembly Mechanisms of Butterfly Communities across Environmental Gradients of a Subtropical Mountain
Source: Insects. 2024 Mar 27;15(4):230. doi: 10.3390/insects15040230 (PMC11050375; doi:10.3390/insects15040230)
Supplement: Supplementary file 1 [file insects-15-00230-s001.zip › supplementary material.pdf]

Supplementary Table S1. Checklist of identified butterflies in the Chebaling study area.

NO.	Family	Genus	Species
1.	Papilionidae	*Papilio*	*Papilio paris*
2.			*Papilio dialis*
3.			*Papilio bianor*
4.			*Papilio protenor*
5.			*Papilio memnon*
6.			*Papilio machaon*
7.			*Papilio xuthus*
8.			*Papilio polytes*
9.			*Papilio helenus*
10.			*Papilio nephelus*
11.		*Graphium*	*Graphium sarpedon*
12.			*Graphium cloanthus*
13.			*Graphium chironides*
14.		*Meandrusa*	*Meandrusa lachinus*
15.	Pieridae	*Eurema*	*Eurema hecabe*
16.			*Eurema blanda*
17.			*Eurema laeta*
18.		*Catopsilia*	*Catopsilia pyranthe*
19.		*Hebomoia*	*Hebomoia glaucippe*
20.		*Ixias*	*Ixias pyrene*
21.		*Leptosia*	*Leptosia nina*
22.		*Talbotia*	*Talbotia naganum*
23.		*Pieris*	*Pieris rapae*
24.			*Pieris canidia*
25.		*Cepora*	*Cepora nadina*
26.			*Cepora nerissa*
27.		*Delias*	*Delias pasithoe*
28.			*Delias acalis*
29.	Lycaenidae	*Taraka*	*Taraka hamada*
30.		*Allotinus*	*Allotinus drumila*
31.		*Miletus*	*Miletus chinensis*
32.		*Curetis*	*Curetis acuta*

33.	*Ravenna*	*Ravenna nivea*
34.	*Leucantigius*	*Leucantigius atayalicus*
35.	*Yamamo*	*Yamamo tozephyrus*
36.	*Arhopala*	*Arhopala ganesa*
37.		*Arhopala paramuta*
38.		*Arhopala bazalus*
39.		*Arhopala rama*
40.	*Mahathaio*	*Mahathaio ameria*
41.	*Catapaecilma*	*Catapaecilma major*
42.	*Tajuria*	*Tajuria maculata*
43.	*Spindasis*	*Spindasis syama*
44.		*Spindasis lohita*
45.	*Sinthusa*	*Sinthusa chandrana*
46.	*Ancema*	*Ancema ctesia*
47.	*Deudorix*	*Deudorix epijarbas*
48.	*Rapala*	*Rapala varuna*
49.		*Rapala micans*
50.		*Rapala manea*
51.	*Heliophorus*	*Heliophorus ila*
52.	*Nacaduba*	*Nacaduba kurava*
53.		*Nacaduba berenice*
54.	*Jamides*	*Jamides bochus*
55.		*Jamides celeno*
56.	*Zizeeria*	*Zizeeria maha*
57.	*Lampides*	*Lampides boeticus*
58.	*Everes*	*Everes argiades*
59.	*Pithecops*	*Pithecops corvus*
60.	*Udara*	*Udara dilecta*
61.		*Udara albocaerulea*
62.	*Acytolepis*	*Acytolepis puspa*
63.	*Celastrina*	*Celastrina argiolus*
64.	*Euchrysops*	*Euchrysops cnejus*
65.	*Chilades*	*Chilades pandava*
66.	*Tongeia*	*Tongeia filicaudis*
67.	*Abisara*	*Abisara fylla*

68.			*Abisara neophron*
69.			*Abisara burnii*
70.			*Abisara echerius*
71.		*Stiboges*	*Stiboges nymphidia*
72.		*Zemeros*	*Zemeros flegyas*
73.		*Dodona*	*Dodona deodata*
74.			*Dodona eugenes*
75.			*Dodona egeon*
76.	Nymphalidae	*Danaus*	*Danaus genutia*
77.		*Parantica*	*Parantica aglea*
78.			*Parantica melaneus*
79.		*Euploea*	*Euploea midamus*
80.		*Acraea*	*Acraea issoria*
81.		*Cethosia*	*Cethosia biblis*
82.		*Argyreus*	*Argyreus hyperbius*
83.		*Damora*	*Damora sagana*
84.		*Argyronome*	*Argyronome laodice*
85.		*Kallima*	*Kallima inachus*
86.		*Hypolimnas*	*Hypolimnas bolina*
87.		*Kaniska*	*Kaniska canacev*
88.		*Polygonia*	*Polygonia c-aureum*
89.		*Junonia*	*Junonia orithya*
90.			*Junonia iphita*
91.			*Junonia almana*
92.		*Symbrenthia*	*Symbrenthia liaea*
93.			*Symbrenthia brabira*
94.			*Symbrenthia hypselis*
95.		*Ariadne*	*Ariadne ariadne*
96.			*Ariadne merione*
97.		*Bhagadatta*	*Bhagadatta austenia*
98.		*Euthalia*	*Euthalia guangdongensi*
99.			*Euthalia kosempona*
100.			*Euthalia omeia*
101.			*Euthalia bunzoi*
102.			*Euthalia pratti*

103.		*Euthalia strephon*
104.		*Euthalia thibetana*
105.		*Euthalia aconthea*
106.		*Euthalia lubentna*
107.		*Euthalia irrubescens*
108.	*Cynitia*	*Cynitia whiteheadi*
109.	*Abrota*	*Abrota ganga*
110.	*Limenitis*	*Limenitis sulpitia*
111.	*Parasarpa*	*Parasarpa dudu*
112.	*Athyma*	*Athyma ranga*
113.		*Athyma cama*
114.		*Athyma selenophora*
115.		*Athyma jina*
116.		*Athyma asura*
117.		*Athyma opalina*
118.		*Athyma zeroca*
119.	*Neptis*	*Neptis kuangtungensis*
120.		*Neptis yerburii*
121.		*Neptis manasa*
122.		*Neptis sankara*
123.		*Neptis philyra*
124.		*Neptis cartica*
125.		*Neptis clinia*
126.		*Neptis soma*
127.		*Neptis miah*
128.		*Neptis hylas*
129.		*Neptis sappho*
130.		*Neptis anantad*
131.	*Phaedyma*	*Phaedyma aspasiav*
132.	*Pantoporia*	*Pantoporia hordonia*
133.	*Stibochiona*	*Stibochiona nicea*
134.	*Dichorragia*	*Dichorragia nesimachus*
135.	*Cyrestis*	*Cyrestis thyodamas*
136.	*Apatura*	*Apatura iliav*
137.	*Rohana*	*Rohana parisatis*

138.	*Helcyra*	*Helcyra superba*
139.		*Helcyra subalba*
140.	*Sephisa*	*Sephisa chandra*
141.	*Sasainia*	*Sasainia funebris*
142.	*Hestina*	*Hestina assimilis*
143.	*Timelaea*	*Timelaea albescens*
144.	*Polyura*	*Polyura narcaea*
145.		*Polyura eudamippus*
146.		*Polyura nepenthes*
147.	*Charaxes*	*Charaxes bernardus*
148.	*Mimathyma*	*Mimathyma chevana*
149.	*Discophora*	*Discophora sondaica*
150.	*Enispe*	*Enispe lunatum*
151.	*Aemona*	*Aemona amathusia*
152.	*Stichophthalma*	*Stichophthalma suffusa*
153.	*Penthema*	*Penthema adelma*
154.	*Neorina*	*Neorina patria*
155.	*Melanitis*	*Melanitis leda*
156.		*Melanitis phedima*
157.	*Lethe*	*Lethe dura*
158.		*Lethe hyrania*
159.		*Lethe chandica*
160.		*Lethe verma*
161.		*Lethe helena*
162.		*Lethe satyrina*
163.		*Lethe confusa*
164.		*Lethe syrcis*
165.		*Lethe europa*
166.		*Lethe mekara*
167.		*Lethe sisii*
168.	*Neope*	*Neope muirheadii*
169.		*Neope bremeri*
170.		*Neope contrasta*
171.	*Mandarinia*	*Mandarinia regalis*
172.	*Mycalesis*	*Mycalesis franciscav*

173.			*Mycalesis mucianus*
174.			*Mycalesis mineus*
175.			*Mycalesis sangaica*
176.			*Mycalesis gotama*
177.		*Ypthima*	*Ypthima multistriata*
178.			*Ypthima imitans*
179.			*Ypthima baldus*
180.			*Ypthima praenubila*
181.			*Ypthima tappana*
182.			*Ypthima motschulskyi*
183.		*Palaeonympha*	*Palaeonympha opalina*
184.		*Hemadara*	*Hemadara narasingha*
185.	Hesperiidae	*Bibasis*	*Bibasis miracula*
186.		*Choaspes*	*Choaspes benjaminii*
187.		*Hasora*	*Hasora vitta*
188.			*Hasora anura*
189.		*Lobocla*	*Lobocla bifasciata*
190.		*Capila*	*Capila pennicillatum*
191.			*Capila translucida*
192.			*Capila lineata*
193.		*Abraximorpha*	*Abraximorpha davidii*
194.		*Abriphasam*	*Abriphasam heringi*
195.		*Celaenorrhinus*	*Celaenorrhinus leucocera*
196.			*Celaenorrhinus vietnamicus*
197.			*Celaenorrhinus maculosus*
198.			*Celaenorrhinus aspersus*
199.			*Celaenorrhinus yaojiani*
200.		*Pseudocoladenia*	*Pseudocoladenia dan*
201.		*Gerosis*	*Gerosis sinica*
202.		*Tagiades*	*Tagiades menaka*
203.			*Tagiades litigiosa*
204.			*Tagiades cohaerens*
205.		*Astictopterus*	*Astictopterus jama*
206.		*Iambrix*	*Iambrix salsala*
207.		*Udaspes*	*Udaspes folus*

208.	*Notocrypta*	*Notocrypta feisthamelii*
209.		*Notocrypta curvifascia*
210.		*Notocrypta paralysos*
211.	*Erionota*	*Erionota torus*
212.	*Matapa*	*Matapa aria*
213.	*Zographetus*	*Zographetus satwa*
214.	*Isoteinon*	*Isoteinon lamprospilus*
215.	*Halpe*	*Halpe gamma*
216.	*Thoressa*	*Thoressa xiaoqingae*
217.	*Ampittia*	*Ampittia virgata*
218.		*Ampittia dioscorides*
219.	*Onryza*	*Onryza maga*
220.	*Ochlodes*	*Ochlodes klapperichii*
221.	*Thymelicus*	*Thymelicus leonius*
222.	*Potanthus*	*Potanthus flavus*
223.		*Potanthus trachalus*
224.		*Potanthus pava*
225.		*Potanthus confucius*
226.	*Telicota*	*Telicota ohara*
227.		*Telicota linna*
228.		*Telicota besta*
229.	*Parnara*	*Parnara ganga*
230.		*Parara guttata*
231.		*Parnara batta*
232.	*Borbo*	*Borbo cinnara*
233.	*Pelopidas*	*Pelopidas assamensis*
234.		*Pelopidas mathias*
235.	*Aeromachus*	*Aeromachus jhora*
236.	*Caltoris*	*Caltoris cahira*
237.		*Caltoris bromus*

Supplementary Table S2. The sampling coverage was estimated using the "iNEXT" package in R across all grids.

NO.	Grid number	Sample coverage
1	A07	0.355
2	A08	0.752
3	B06	0.640
4	B07	0.644
5	B08	0.894
6	C06	0.542
7	C07	0.781
8	C08	0.829
9	C09	0.667
10	D05	0.815
11	D06	0.840
12	D07	0.864
13	D08	0.900
14	D09	0.578
15	E05	0.521
16	E06	0.660
17	E07	0.854
18	E08	0.828
19	E09	0.794
20	E10	0.606
21	E11	0.835
22	F03	0.558
23	F04	0.618
24	F05	0.721
25	F06	0.750
26	F07	0.742
27	F08	0.342
28	F09	0.590
29	F10	0.605
30	G02	0.572
31	G03	0.828
32	G04	0.754
33	G05	0.725
34	G06	0.612
35	G07	0.773
36	G08	0.874
37	G09	0.579
38	G10	0.727
39	H01	0.928
40	H02	0.720

41	H03	0.862
42	H04	0.694
43	H05	0.936
44	H06	0.759
45	H07	0.773
46	H08	0.776
47	H09	0.548
48	I03	0.765
49	I04	0.704
50	I05	0.877
51	I06	0.707
52	I07	0.655
53	I08	0.795
54	I09	0.612
55	J03	0.571
56	J04	0.807
57	J05	0.725
58	J06	0.927
59	J07	0.834
60	J08	0.903
61	J09	0.624
62	K05	0.527
63	K06	0.410
64	K07	0.867
65	K08	0.601
66	K09	0.611
67	K10	0.744
68	L05	0.652
69	L06	0.632
70	L07	0.492
71	L08	0.560
72	M06	0.873
73	M07	0.702
74	M08	0.697
75	N06	0.852
76	N07	0.731
77	N08	0.504
78	O06	0.795
79	O07	0.822
80	P05	0.790

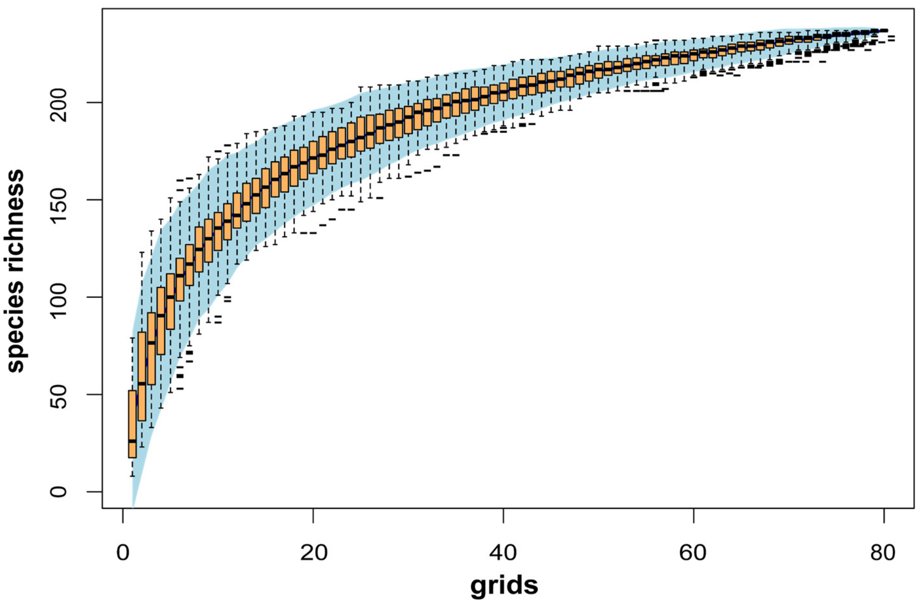

Supplementary Figure S1. Species accumulation curves of the observed butterfly samples in Chebaling.

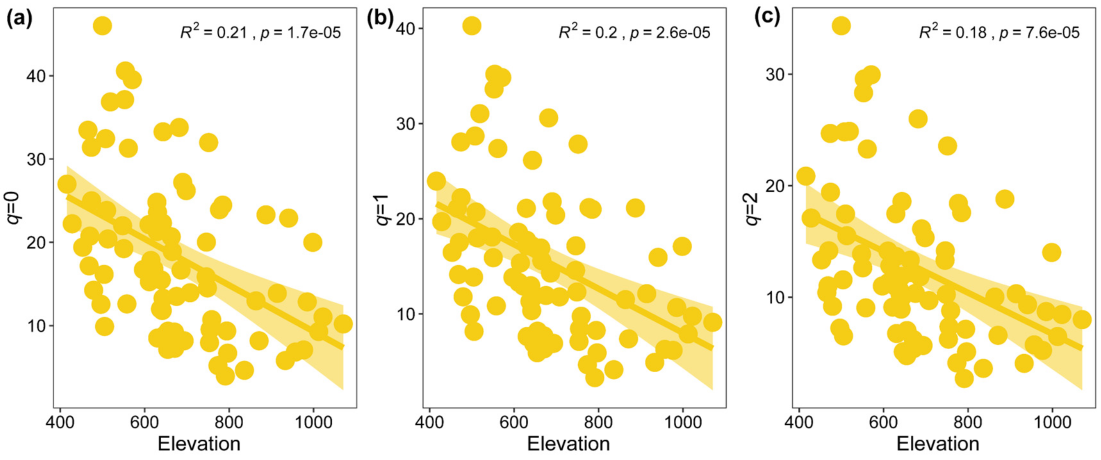

Supplementary Figure S2. Variations of (a) Hill numbers (q = 0), (b) Hill numbers (q = 1), and (c) Hill numbers (q = 2) of butterflies along elevation in Chebaling, regression lines refer to the significant relationship between the two variables detected through linear models and shading areas associated with the lines represent the 95% confidence interval.

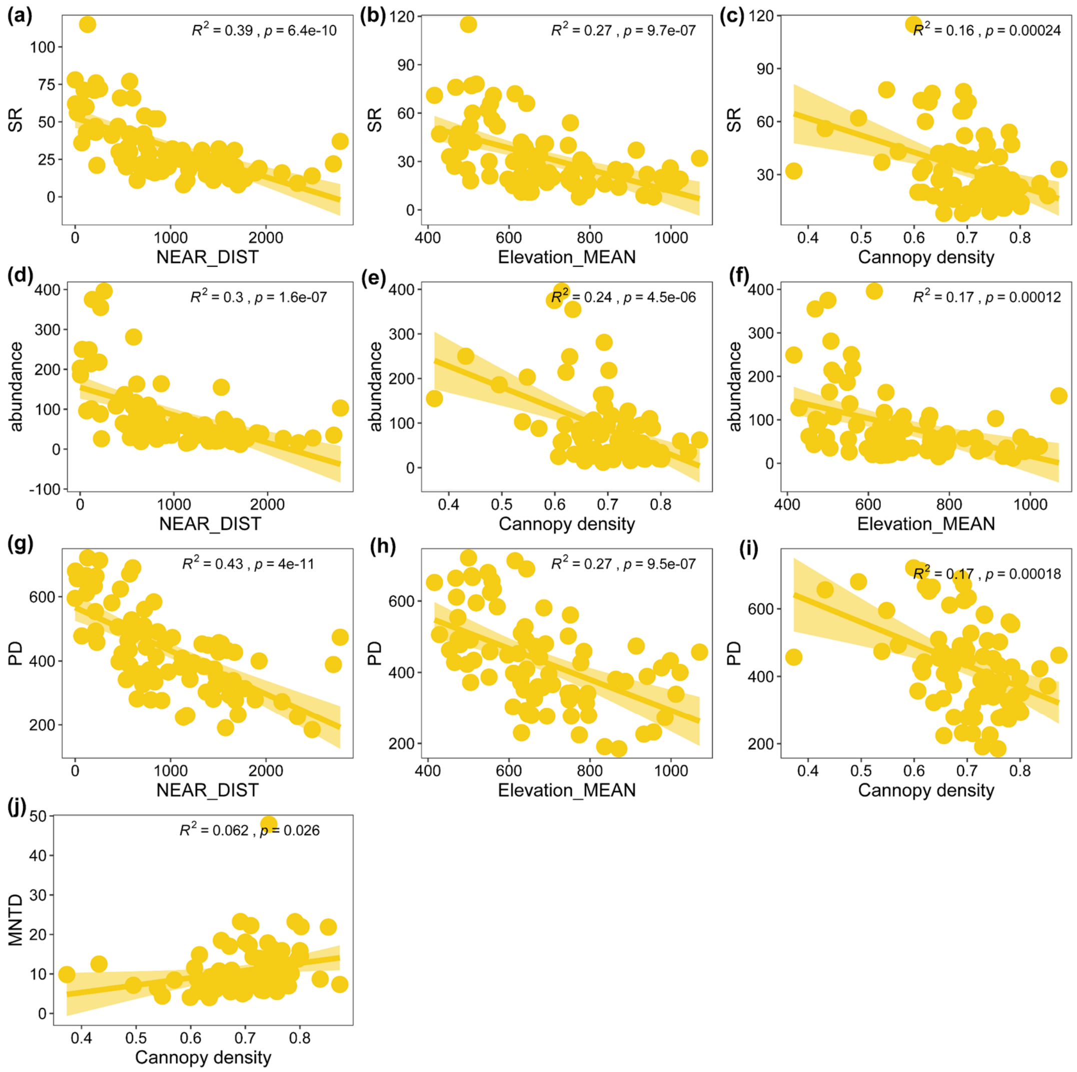

Supplementary Figure S3. Correlation between diversity indices and environmental factors (the most important drivers analyzed by Random Forest model). Relationships between NEAR_DIST and (a) species richness (SR), (d) abundance, (g) Faith's phylogenetic diversity (PD), and (j) mean nearest taxon distance (MNTD); relationships between Elevation_MEAN and (b) SR, (f) abundance, and (h) PD; relationships between Cannopy density and (c) SR, (e) abundance, and (i) PD. Regression lines refer to the significant relationship between the two variables detected through linear models and shading areas associated with the lines represent the 95% confidence interval.

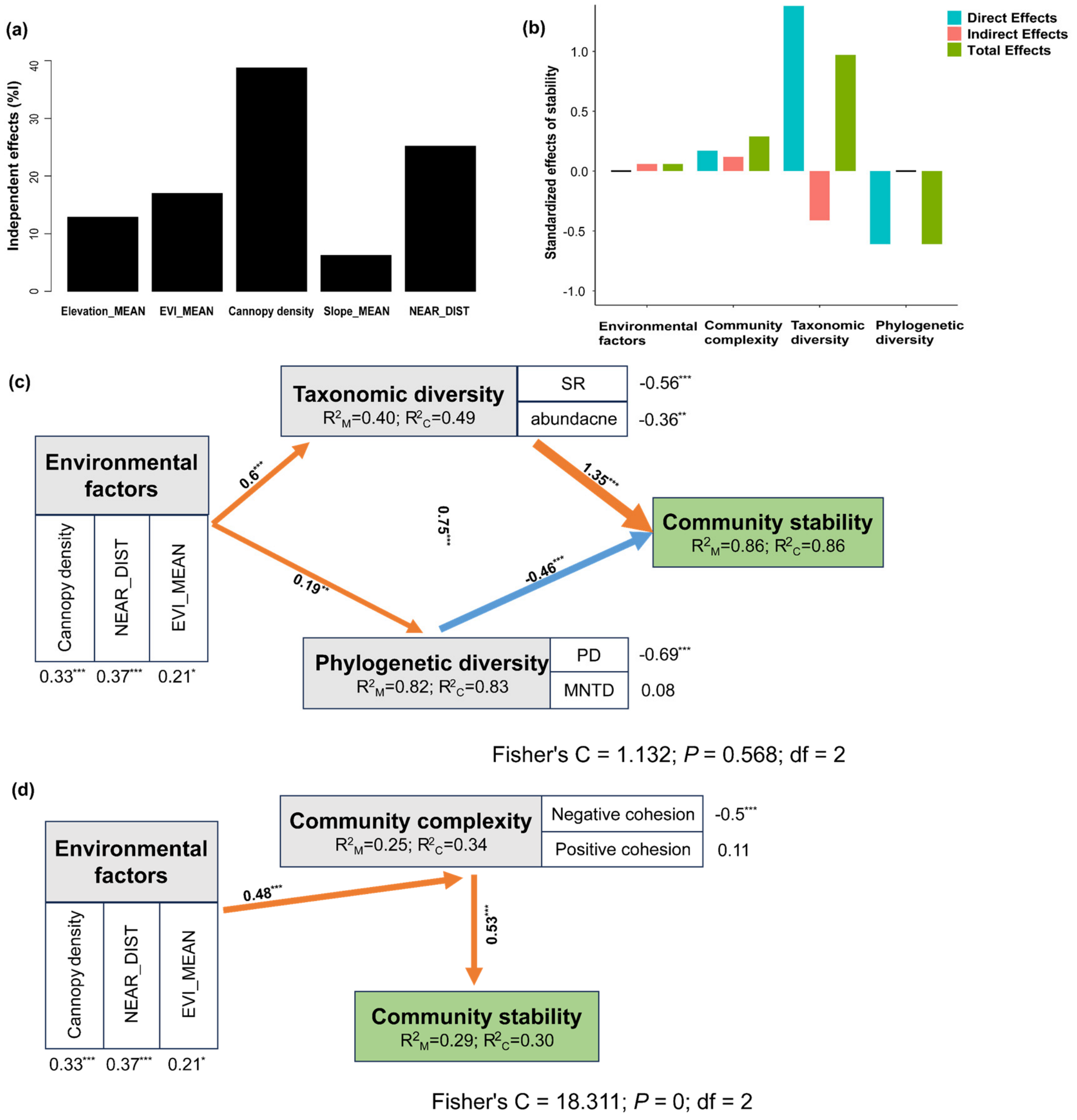

Supplementary Figure S4. (a) Visualization of the independent contributions of the different environmental factors to the community stability (result of R-function "hier.part"); (b) the direct, indirect, and total standardized effects of composite variables on community stability; (c) the direct and indirect effects of environmental factors, taxonomic diversity, and phylogenetic diversity on the responses of community stability using piecewiseSEM; (d) the direct and indirect effects of environmental factors and community complexity on the responses of community stability using piecewiseSEM. The environmental factors, community complexity, taxonomic diversity, and phylogenetic diversity were altered to composite variables. Numbers adjacent to measured variables are their coefficients with composite variables. Numbers adjacent to arrows are path coefficients are the directly standardized effect size of the relationship. The thickness of the arrow represents the strength of the relationship. The conditional (C) and marginal (M) R^2 represent the proportion of variance explained by all predictors without and with accounting for random effects of "sampling site". Significance levels of each predictor are $*p < 0.05$, $**p < 0.01$, $***p < 0.001$.

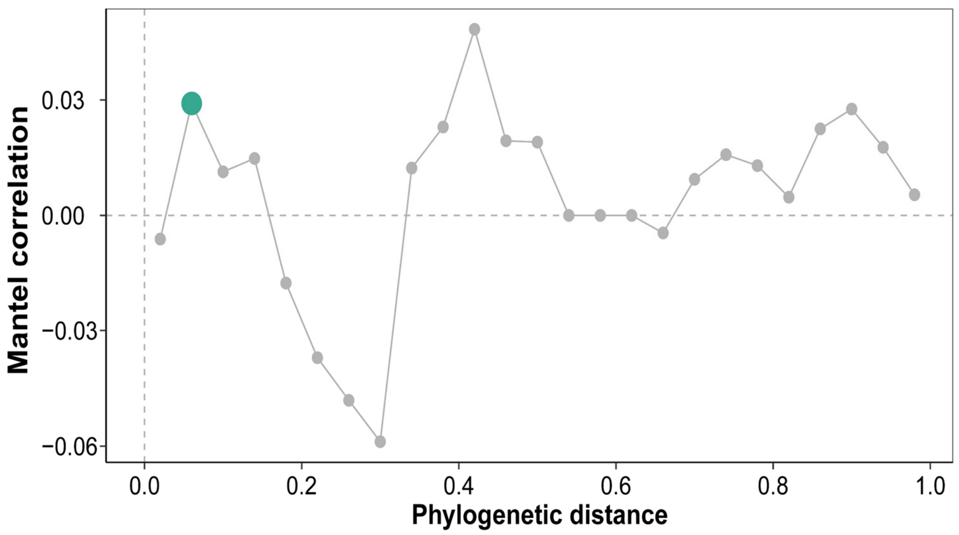

Supplementary Figure S5. Phylogenetic Mantel correlogram evaluating phylogenetic signal in the butterfly communities observed in this study. The plot relates Pearson correlation coefficients to phylogenetic distances classes. Significant correlations (P < 0.05; green dots) indicate significant phylogenetic signal but only across relatively short phylogenetic distances.

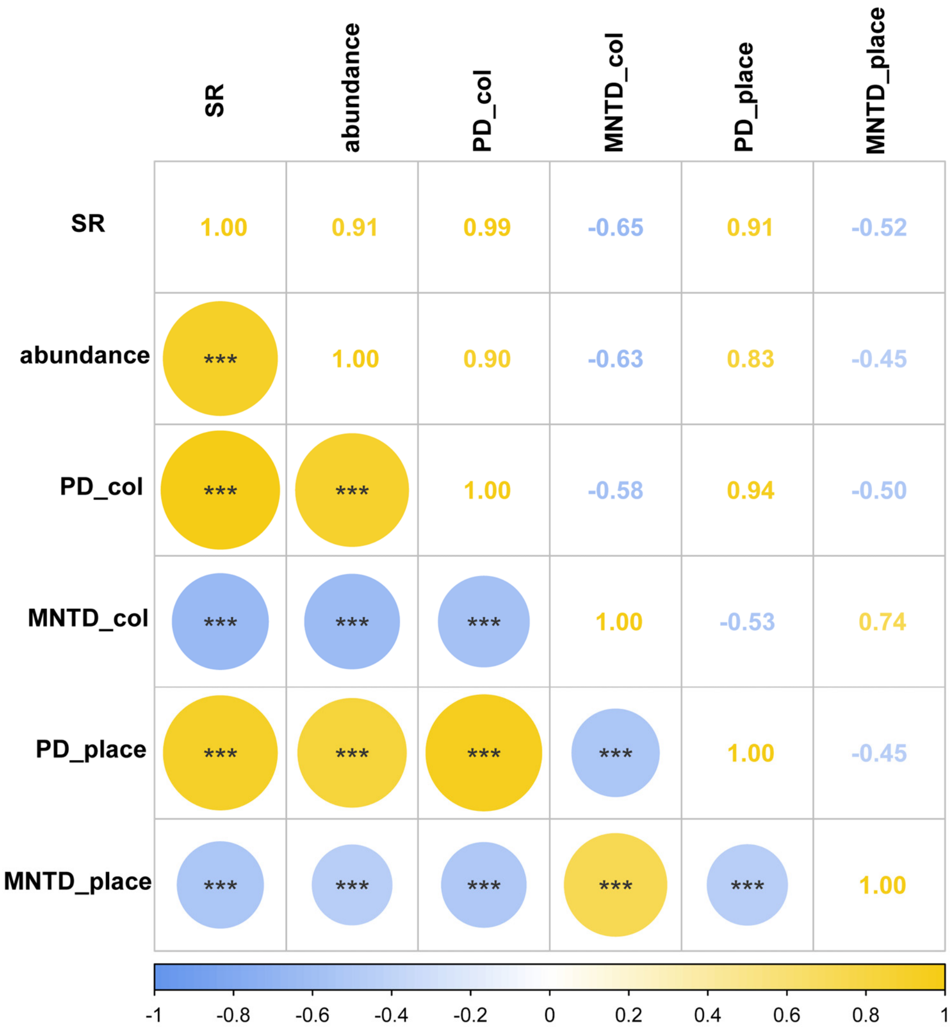

Supplementary Figure S6. Correlation among species richness (SR), abundance, Faith's phylogenetic diversity (PD), and mean nearest taxon distance (MNTD). PD_col and MNTD_col were calculated using the barcode-tree; PD_place and MNTD_place were calculated using the placement tree. Significance signs for correlation coefficients are marked in the circles: * means $p < 0.05$, ** means $p < 0.01$, *** means $p < 0.001$.
